# Spontaneous remission in primary membranous nephropathy: mechanisms, predictive factors, and implications for personalized management

**DOI:** 10.3389/fimmu.2025.1651810

**Published:** 2025-09-11

**Authors:** Mengqi Wu, Yanhao Chen, Zixin He, Youping Jin

**Affiliations:** ^1^ Department of Nephrology, Hangzhou Traditional Chinese Medicine Hospital of Zhejiang Chinese Medical University, Hangzhou, Zhejiang, China; ^2^ The Second School of Clinical Medicine, Zhejiang Chinese Medical University, Hangzhou, Zhejiang, China; ^3^ Department of Respiratory Internal Medicine, Lishui Hospital of Traditional Chinese Medicine Affiliated to Zhejiang Chinese Medical University, Lishui, Zhejiang, China; ^4^ Department of Nephrology, Lishui Hospital of Traditional Chinese Medicine Affiliated to Zhejiang Chinese Medical University, Lishui, Zhejiang, China

**Keywords:** primary membranous nephropathy, spontaneous remission, anti-PLA2R antibody, risk stratification, precision therapy

## Abstract

Primary membranous nephropathy (PMN) is a major cause of adult nephrotic syndrome and demonstrates considerable clinical heterogeneity. This review summarizes current evidence on the immunological mechanisms and clinical predictors underlying spontaneous remission (SR) in PMN. We discuss key factors including the dynamics of anti-PLA2R antibodies, proteinuria trends, renal function indicators, histopathological features, and emerging biomarkers. A staged immune modulation process is proposed, involving suppression of autoreactive responses and promotion of tissue repair. Integrating these insights, we also outline a personalized treatment approach based on dynamic risk stratification and longitudinal monitoring. Understanding the drivers of SR may help reduce unnecessary immunosuppression and guide precision management in PMN.

## 1 Introduction

Membranous nephropathy (MN) is a glomerular disease characterized by diffuse thickening of the glomerular basement membrane (GBM) (1, 2). It predominantly affects middle-aged and elderly individuals and represents one of the most common causes of nephrotic syndrome in adults (3, 4). The global incidence of MN is estimated at 8–10 cases per million population (3, 5). In China, MN accounts for 23.4% of biopsy-proven glomerular diseases, second only to IgA nephropathy, with a steadily increasing incidence over recent years (6). MN can be classified into primary and secondary forms, with primary MN (PMN) accounting for approximately 80% of cases (7). A major milestone in understanding MN pathogenesis was achieved in 2009, when Beck et al. identified the M-type phospholipase A2 receptor (PLA2R), located on podocyte membranes, as the predominant target antigen in idiopathic MN, along with its corresponding autoantibody (8). This discovery established MN as an autoimmune disease and significantly transformed both diagnostic and monitoring strategies. Subsequently, other target antigens, such as THSD7A, have also been identified (9, 10).

With growing insights into its pathogenesis, the diagnosis and treatment of MN have evolved toward more specific and effective approaches. Currently, immunosuppressive therapy, anti-CD20 monoclonal antibodies, and other novel therapies are the mainstay of clinical management (11). Notably, MN exhibits a highly heterogeneous clinical course, ranging from spontaneous remission (SR) to progression to end-stage kidney disease (ESKD). Approximately one-third of patients with PMN exhibit a benign or indolent disease course, with a SR rate of up to 30% (12). Another one-third of patients develop nephrotic syndrome while maintaining preserved renal function. Nonetheless, 15–50% of untreated patients progress to ESKD within 10 years (19, 20). SR is therefore an important clinical phenomenon in MN, with substantial implications for long-term prognosis and therapeutic decision-making. The occurrence of SR indicates that some patients may improve without the need for intensive immunosuppressive therapy, thereby avoiding the adverse effects associated with such treatments (13). However, due to the substantial proportion of patients who exhibit persistent disease activity or progression, the optimal timing to initiate immunosuppressive therapy remains controversial (14).

The KDIGO 2012 guideline took a conservative approach—it recommended up to 6 months of maximal supportive care before starting immunosuppressive therapy, in order to allow for potential SR. Immunosuppression was only advised for patients who remained at high risk of progression (15). This strategy, while avoiding unnecessary drug toxicity, had limited predictive accuracy—nearly half of patients meeting the high-risk criteria at 6 months may still go on to achieve SR without immunosuppressive therapy (16). In light of emerging prognostic markers, the KDIGO 2021 guideline introduced a more individualized, risk-stratified approach to managing primary MN (17). Patients are now categorized into low, moderate, high, or very high risk groups based on a combination of clinical and biomarker features rather than time-dependent proteinuria alone. Besides the degree of proteinuria and trend in renal function, the 2021 criteria incorporate serum albumin levels, PLA2R antibody titers, and even urinary biomarkers to refine risk assessment (18). According to the updated recommendations, patients in high-risk or very high-risk categories are advised to initiate immunosuppressive therapy promptly, rather than waiting a full six months, given their elevated risk of progressive kidney injury. In particular, individuals with persistently nephrotic-range proteinuria or with declining renal function are now considered for early intervention (18). This proactive strategy aims to prevent irreversible damage in those unlikely to undergo SR.

Crucially, even under the new paradigm, clinicians must recognize that some patients predicted to be “high risk” can still achieve SR. There are documented cases of very high-risk MN patients (with massive proteinuria and even complications like thrombosis) who have improved with conservative management alone (19). Such observations underscore the dynamic nature of MN: risk status at diagnosis is not a perfect predictor of disease trajectory. Therefore, current guidelines emphasize continual re-assessment and a personalized treatment approach. Careful monitoring of disease markers is recommended to guide if and when to escalate therapy. A thorough understanding of the mechanisms and determinants of SR in MN may help clinicians identify patients who are suitable for conservative management and those who require timely therapeutic intervention, ultimately facilitating personalized and precision medicine. This review summarizes the latest advances in the study of SR in MN, including mechanistic insights, clinical data and predictive factors, management strategies, and future research directions, aiming to provide a reference for clinical practice and further investigation.

## 2 Mechanisms of spontaneous remission

### 2.1 Immunological insights

The immunopathogenesis of PMN has been extensively elucidated. As the principal target cells in the immune response, podocytes serve as sites of injury by providing endogenous antigens or by creating a microenvironment that favors antigen deposition (20). In PMN, autoimmune injury is primarily mediated by autoantibodies that recognize target antigens on podocytes, leading to the formation of immune complexes beneath the foot processes and on the outer aspect of the GBM. These immune complexes activate the complement cascade and cause podocyte injury, resulting in increased GBM permeability and massive proteinuria, thereby initiating disease progression (21). However, not all MN patients exhibit progressive disease; a significant proportion experience spontaneous attenuation or cessation of the autoimmune response, leading to SR. Although the precise immunoregulatory mechanisms underlying SR remain unclear (22), clinical mechanistic data remain limited, as these low-risk patients have traditionally not been extensively studied. Nevertheless, biospecimen research in this population may yield valuable mechanistic insights into SR and help inform the development of more targeted and safer therapeutic strategies in MN. Several possible pathways have been proposed. Rosenzwajg et al. demonstrated the involvement of both B and T lymphocytes in the pathogenesis of MN (23). One hypothesis suggests that B lymphocytes possess an intrinsic self-limiting function. B cells differentiate into plasma cells, which produce anti-PLA2R antibodies targeting antigens on podocyte membranes (23). In some patients, the B cell clones responsible for anti-PLA2R antibody production may undergo exhaustion or be eliminated through immune surveillance over time, resulting in a spontaneous decline and eventual disappearance of antibody titers. A meta-analysis by Zhang J et al. further confirmed the predictive value of anti-PLA2R antibody levels for SR in idiopathic MN (IMN); lower baseline anti-PLA2R titers were associated with a higher likelihood of SR (24). In line with these findings, recent studies have shown that patients who achieve SR tend to have a less aggressive clone of autoreactive B cells and a more favorable immunoregulatory profile, including a recovery of regulatory B cells (Bregs) and regulatory T cells (Tregs) that are reduced during active disease but restored in remission (25). Restoration of immune tolerance in this context is thought to be mediated primarily through Tregs and potentially Bregs, which can suppress pathogenic immune responses and promote the re-establishment of immune homeostasis (26–28). In addition to the restoration of conventional CD4^+^ Tregs, recent experimental work has highlighted the contribution of CD8^+^ Tregs in MN immune regulation. Animal models of MN (Heymann nephritis) demonstrate a role for CD8^+^ T cells in down-regulating autoimmunity and attenuating disease. Classic adoptive-transfer studies identified antigen-specific OX8^+^ (CD8^+^) suppressor T cells that re-establish tolerance late in active Heymann’s nephritis (29). More recent work shows that CD8^+^ Tregs, induced by T-cell or peptide vaccination, limit autoantibody production and kidney injury by Qa-1 (HLA-E)–restricted elimination of autoreactive CD4^+^ T-follicular helper cells (30, 31). In humans, an analogous KIR^+^ CD8^+^ regulatory subset can delete pathogenic CD4^+^ T cells ex vivo across several autoimmune diseases (32), although its role in PMN remains to be defined. Together with Bregs and CD4^+^ Tregs, these CD8^+^ Tregs may provide an additional layer of immune regulation contributing to SR in MN.

Such immune tolerance mechanisms underscore the interplay between cellular and humoral immunity in MN, as T cells play a crucial role in supporting B cell-mediated antibody production. Enhanced activity of Tregs or suppression of pro-inflammatory T cells, such as follicular helper T cells (T_FH), could diminish B cell function. Clinical observations of disease remission following infections in some MN patients support the possibility of immune state reprogramming (33–35). It has been hypothesized that widespread immune activation induced by infection may paradoxically “reset” immune homeostasis and facilitate the disappearance of autoantibodies (23, 33). In addition, feedback regulation from the innate immune system may also contribute to SR. Ongoing immune complex deposition and podocyte injury might elicit negative feedback signals that attenuate immune responses. For example, anti-inflammatory cytokines secreted by macrophages during clearance of immune deposits or the upregulation of inhibitory co-stimulatory molecules such as PD-L1 on injured podocytes (36) may play roles in suppressing the autoimmune response. Nevertheless, these proposed mechanisms remain speculative and require further experimental validation.

### 2.2 Dynamics of anti-PLA2R antibodies

In PMN, approximately 70%–80% of patients have detectable circulating autoantibodies against the M-type PLA2R, predominantly of the IgG4 subclass (8). Serial monitoring of anti-PLA2R antibody levels has become an essential tool in disease assessment and offers important insights into the mechanisms of SR. Antibody titers closely correlate with disease activity, with elevated levels generally reflecting ongoing immune-mediated glomerular injury (37, 38). Clinically, a decline or seroconversion of anti-PLA2R antibody titers is often observed several months prior to the onset of SR (39–41). Studies on rituximab treatment in MN further support a temporal lag between antibody clearance and proteinuria resolution; whether antibody reduction is treatment-induced or occurs spontaneously, the improvement in proteinuria typically follows 3–6 months later (42). This temporal sequence suggests that a reduction in anti-PLA2R antibodies—whether through immunosuppressive treatment or intrinsic immune regulation—leads to a gradual cessation of new immune injury to the glomeruli. Subsequently, existing lesions enter a reparative phase, manifesting clinically as progressive reduction in proteinuria and eventual remission (42). In spontaneously remitting patients, the decline in antibody titers occurs endogenously.

Notably, baseline anti-PLA2R antibody levels are strongly predictive of disease course: patients with low titers are more likely to achieve SR, while those with high titers are less likely to do so. It has been reported that among patients with anti-PLA2R titers in the highest tertile, the probability of spontaneous immunologic remission (i.e., antibody clearance) under supportive care alone is as low as 4% (43–46). In contrast, patients with lower antibody levels may benefit from extended observation, allowing for the possibility of natural resolution of immune activity. In addition to PLA2R, anti-THSD7A antibodies are present in approximately 2.5%–5% of patients with primary MN and have similar pathogenic mechanisms (47). Although the SR rate of THSD7A-associated MN remains unclear, the frequent coexistence of malignancies in such cases suggests that resolution of the underlying tumor may contribute to disease remission (48). Beyond PLA2R and THSD7A, recent studies have identified other pathogenic podocyte antigens that together account for up to 20% of PMN cases, including neural epidermal growth factor-like 1 protein (NELL-1) (49), semaphorin-3B (SEMA3B) (50), protocadherin 7 (PCDH7) (51), and exostosin 1/2 (EXT1/2) (52). NELL-1, a secreted extracellular matrix–associated glycoprotein, accounts for ~5–10% of primary cases and is often detected in older adults, frequently in the context of malignancy. Histologically, NELL-1–associated MN is characterized by segmental or global subepithelial deposits with an IgG1-dominant pattern, and in paraneoplastic cases, tumor removal has been associated with disappearance of circulating antibodies and subsequent remission (49, 53). Although long-term data on SR in these non-PLA2R subtypes are limited, available evidence indicates that dynamic declines in their respective autoantibody levels—particularly following removal of an underlying trigger—may parallel the immunologic–clinical remission sequence observed in PLA2R-associated MN. Overall, dynamic monitoring of autoantibodies provides a critical window into the immune activity of MN. SR is often preceded by a natural decline in pathogenic antibody levels, likely reflecting a re-establishment of immune homeostasis.

### 2.3 Pathological repair processes

In MN, podocyte injury and subepithelial immune complex deposition are the primary causes of proteinuria. During SR, the attenuation of immune activity enables the glomeruli to initiate intrinsic repair processes. Ultrastructural studies have revealed a progression of pathological stages over time: early lesions are characterized by abundant subepithelial electron-dense deposits; in the intermediate phase, these deposits are enveloped by newly formed basement membrane material with characteristic “spikes” visible on silver staining; in later stages (Stage IV), deposits may be partially resorbed, dispersed, or calcified (54).

Renal biopsies from patients undergoing spontaneous or treatment-induced remission often show reduced or absent immune deposits, as well as residual “holes,” suggesting degradation or clearance of previously formed immune complexes and partial restoration of GBM integrity (54). This clearance is thought to be mediated by phagocytic activity of mesangial cells or infiltrating macrophages. Recent studies indicate that podocyte recovery during SR involves reassembly of slit diaphragm components such as nephrin and podocin, together with reorganization of the actin cytoskeleton (55). These molecular events are closely linked to the recovery of endothelial–podocyte crosstalk, which is critical for maintaining glomerular filtration integrity (56, 57). Furthermore, following resolution of injury, podocytes can partially recover their microvillus architecture, and foot process effacement may reverse, thereby restoring selective permeability of the filtration barrier. Importantly, SR is typically a gradual process. Even after cessation of new immune deposition, the clearance of existing subepithelial deposits and recovery of podocyte function may take months or even years (58). Clinically, this is reflected in a stepwise decline in proteinuria—from nephrotic-range levels to partial remission, and eventually to complete or near-complete remission (58). Restoration of the glomerular filtration barrier and nephron function is central to this process. It is worth noting that the capacity for SR also depends on the reversibility of the lesions and the compensatory function of remaining nephrons. In cases where irreversible basement membrane scarring or tubulointerstitial fibrosis has occurred, proteinuria may persist at a residual level despite immunologic quiescence. Therefore, the potential for SR is ultimately influenced by the extent of structural reversibility and renal reserve.

**Figure 1:**
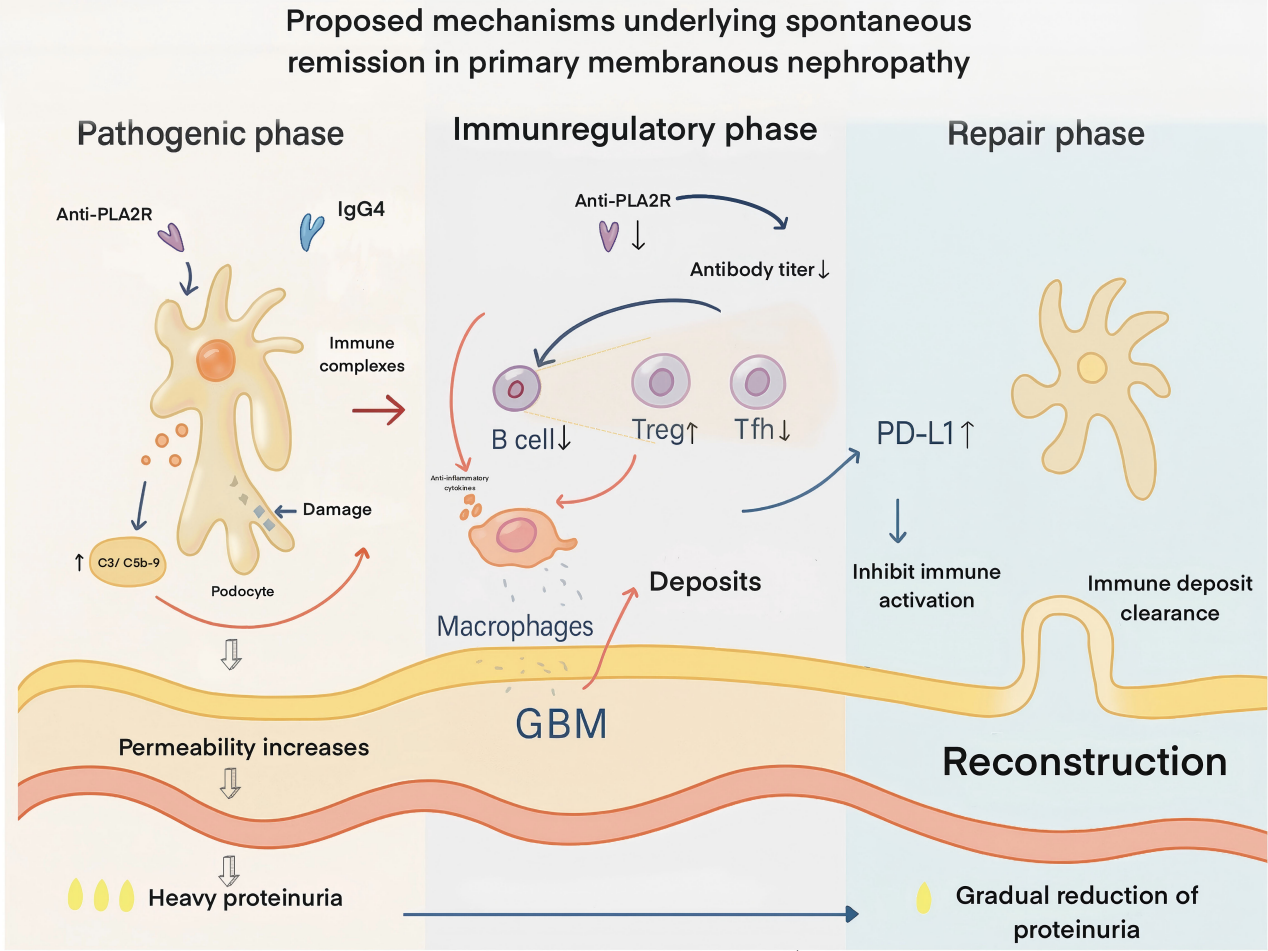
Proposed mechanisms underlying spontaneous remission in primary membranous nephropathy. In the pathogenic phase (left), IgG4 anti-PLA2R autoantibodies bind podocyte antigens, forming subepithelial immune complexes that activate complement (C3, C5b-9), injure podocytes, increase glomerular basement membrane (GBM) permeability, and cause heavy proteinuria. In the immunoregulatory phase (middle), autoantibody titers decline due to reduced pathogenic B-cell activity, enhanced regulatory T-cell (Treg) function, suppression of T follicular helper (T_FH) cells, and macrophage-mediated anti-inflammatory effects. Podocyte PD-L1 upregulation inhibits local immune activation, leading to cessation of new deposit formation. In the repair phase (right), immune deposits are progressively cleared, podocyte architecture and slit diaphragm integrity are restored, GBM structure is reconstructed, and proteinuria gradually resolves. *PLA2R – phospholipase A2 receptor; GBM – glomerular basement membrane; Treg – regulatory T cell; T_FH – T follicular helper T cell; PD-1/PD-L1 – programmed death-1 and its ligand (an immune checkpoint pathway)*.

Therefore, most prevailing theories support that SR in MN is a process of gradually attenuating immune activity. This process is underpinned by a reduction or cessation in the production of pathogenic autoantibodies, clearance of immune deposits, restoration of podocyte structural and functional integrity, and the synergistic involvement of multi-level immunoregulatory mechanisms. These insights help explain why a subset of MN patients can achieve clinical improvement without the need for immunosuppressive therapy (**Figure 1**).

## 3 Predictors of spontaneous remission in PMN

**Figure 2:**
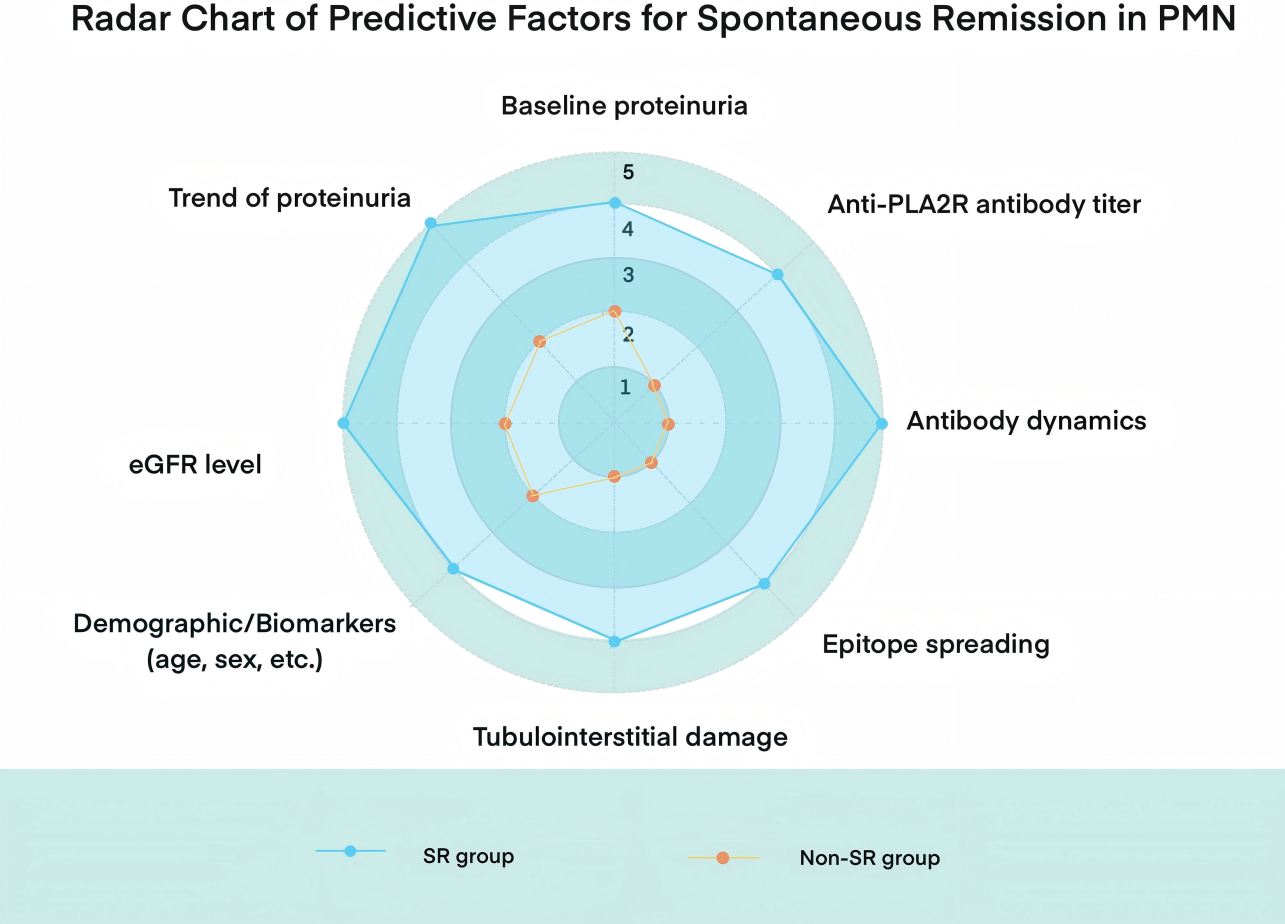
Radar chart comparing key clinical and immunological predictors between spontaneous remission (SR) and non-spontaneous remission (non-SR) groups in PMN.

Identifying factors that influence the likelihood of SR in MN has long been a key research focus. Based on cumulative evidence from previous studies, several predictors of SR have been proposed (**Figure 2**):

### 3.1 Baseline proteinuria and its temporal trajectory

Proteinuria level is the most direct clinical indicator. Classic natural history studies have shown that higher baseline proteinuria and more severe nephrotic syndrome are associated with a lower likelihood of SR (59, 60), a finding that has been consistently validated in more recent investigations (61–64). Patients with subnephrotic proteinuria generally have an excellent prognosis, with 10-year renal survival rates approaching 100%. Many of these individuals can achieve SR even without immunosuppressive therapy (65). In general, the greater the baseline proteinuria, the lower the probability of SR. Although it is traditionally believed that patients with proteinuria >8 g/day rarely achieve SR (66, 67), recent large cohort studies have challenged this notion. These studies demonstrate that even among patients with very high proteinuria, a substantial proportion—nearly one-quarter—achieved remission following intensive supportive therapy. The same study also found that for every 1 g/day reduction in baseline proteinuria, the probability of remission increased accordingly (12). Thus, the overall trend remains: patients with lower baseline proteinuria are more likely to experience SR. As such, baseline proteinuria can serve as a basis for risk stratification.

Moreover, monitoring the trajectory of proteinuria over time is critical for distinguishing progressive disease from self-limited courses. If proteinuria shows a sustained decline under optimized supportive therapy, the patient is likely entering a remission pathway. In such cases, continued conservative management may allow SR to occur without immunosuppression. Conversely, the absence of improvement—or worsening proteinuria—within the first six months suggests ongoing disease activity and low likelihood of SR, warranting consideration of immunosuppressive therapy. According to the 2012 KDIGO guideline for idiopathic MN, initiation of immunosuppressive treatment is recommended when proteinuria exceeds 4 g/day, remains above 50% of baseline, and shows no progressive decline after six months of supportive care. Troyanov et al. proposed a practical rule: failure to achieve a ≥25% reduction in proteinuria at six months should be regarded as treatment failure and prompt a reassessment of therapeutic strategy (12). These dynamic criteria have been increasingly integrated into clinical decision-making to guide individualized treatment planning.

### 3.2 Anti-PLA2R antibody titers and their dynamic changes

Anti-PLA2R antibody titers represent one of the core biomarkers in prognostic evaluation of PMN. Recent meta-analyses suggest that baseline serum anti-PLA2R antibody levels are closely associated with the likelihood of clinical remission (68). Multiple studies have demonstrated that high antibody titers are associated with delayed or absent remission, whereas low titers predict a higher probability of SR (69–72). In a prospective cohort of 65 PMN patients, Jurubi^-^ă et al. used multivariate Cox regression and found that patients who were seronegative for anti-PLA2R at baseline had a significantly higher chance of achieving SR (hazard ratio ~3), indicating that serological status is an independent predictor (73). An updated systematic review that included 18 studies further confirmed that patients with high baseline anti-PLA2R titers had significantly lower clinical remission rates, with this association being particularly strong in Asian populations (24). Despite ongoing efforts to define an optimal anti-PLA2R threshold for predicting SR, there is currently no consensus regarding the ideal cutoff value, value range, or the most informative time points for measurement (39). Some earlier studies observed that patients with titers in the highest tertile or absolute levels >275 U/mL had only a ~20% chance of SR (71). More recently, a retrospective cohort study found that a titer <40 U/mL at diagnosis strongly predicted SR within the first 6 months (67). However, heterogeneity in immunologic and clinical practice standards between institutions makes it difficult to draw definitive conclusions regarding the predictive accuracy of any specific threshold (40, 74). It is important to note that antibody titers often correlate with the severity of proteinuria. Patients with higher titers tend to have more severe disease and are more likely to receive immunosuppressive therapy (75, 76), which may confound the evaluation of purely “spontaneous” remission.

Dynamic changes in anti-PLA2R titers are also important in predicting remission. Generally, a decrease or seroconversion of antibody titers precedes the onset of clinical proteinuria remission. Studies have shown that in nearly all patients, disappearance of anti-PLA2R antibodies precedes complete proteinuria remission by several weeks to months (42). A prospective study by Jatem-Escalante et al. demonstrated that a baseline titer ≤97.5 RU/mL and a ≥15% decline in anti-PLA2R titer within the first 3 months after diagnosis could predict a ≥50% reduction in proteinuria by 6 months (39). Thus, a sustained and significant decrease or seroconversion in antibody titers during observation strongly suggests that the patient is on a path toward SR. Conversely, persistently high anti-PLA2R titers indicate a low likelihood of SR.

### 3.3 Epitope spreading of the anti-PLA2R antibody response

Epitope spreading refers to the broadening of antibody reactivity to multiple epitopes of a given antigen. In PLA2R-associated MN, anti-PLA2R antibodies primarily target the N-terminal cysteine-rich (CysR) domain. However, in some patients, the antibody response “spreads” to include additional epitopes such as CTLD1 and CTLD7 domains. Some studies have suggested that patients with antibodies restricted to the CysR epitope are more likely to achieve SR, whereas those with epitope spreading exhibit more severe proteinuria and poorer outcomes (70). A French study involving 48 PLA2R-positive PMN patients reported that none of the patients with baseline epitope spreading achieved SR, in stark contrast to those without spreading (70). However, this concept has recently been challenged. Reinhard et al., in a long-term follow-up study of 150 PLA2R-positive patients, found that nearly all patients exhibited broad epitope reactivity at diagnosis. Their multivariate analysis showed that overall antibody titer levels, rather than epitope distribution, were more predictive of remission outcomes (77). Thus, the prognostic value of epitope spreading as an independent predictor remains inconclusive. In addition, a lack of standardized methods for epitope mapping poses challenges for routine clinical application. At present, dynamic quantification of anti-PLA2R antibody levels remains the primary approach for guiding clinical judgment.

### 3.4 Renal function indicators

Glomerular filtration rate (eGFR) and serum creatinine levels reflect the degree of renal parenchymal injury. In general, preserved renal function at diagnosis (i.e., normal or mildly reduced eGFR) is associated with a higher likelihood of SR. A retrospective study stratified by baseline eGFR found that patients with eGFR >60 mL/min/1.73 m² had significantly higher remission rates than those with lower eGFR (78). Data from Polanco et al. also demonstrated that lower baseline serum creatinine is an independent predictor of SR (12). Intact kidney function indicates the absence of substantial irreversible damage, suggesting a greater potential for functional recovery once immune deposits are cleared. It is noteworthy that some PMN patients may experience acute functional deterioration during the disease course, such as a sudden rise in serum creatinine, often due to severe hypoperfusion, renal vein thrombosis, or concurrent interstitial pathology. A recent study from China by Li et al. retrospectively reviewed 136 PMN patients and found that 24.1% had experienced acute kidney injury (AKI), with a significantly lower rate of complete SR compared to those without AKI (78). Renal biopsies in these patients often revealed acute tubular injury and extensive interstitial damage, suggesting that an episode of AKI may impose additional irreversible injury, thereby diminishing the likelihood of SR (79). In that study, the AKI group had slightly higher baseline proteinuria than the non-AKI group, but the difference was not statistically significant. Interestingly, anti-PLA2R antibody positivity was significantly lower in the AKI group, and neither proteinuria nor anti-PLA2R titer was identified as an independent predictor of AKI after multivariate adjustment. This suggests that the association between AKI and lower SR rates is not simply explained by higher proteinuria or antibody levels, and that AKI itself may be an independent marker of poor prognosis. Thus, a history of AKI may serve as a useful parameter in risk stratification: its presence indicates a poorer prognosis and warrants more proactive treatment rather than prolonged observation.

### 3.5 Histopathological findings

The degree of tubulointerstitial damage (TID) observed on renal biopsy is one of the most powerful histologic predictors of MN prognosis (80). In 2019, Maria J. Stangou et al. reported that glomerulosclerosis and tubulointerstitial injury in PMN patients were positively correlated with the severity of proteinuria and served as independent determinants of renal function impairment (81). More recent studies have further confirmed this association. Sun et al. found that PMN patients with greater degrees of chronic TID had significantly lower SR rates and a higher likelihood of progressing to renal insufficiency (79). Multivariate analysis indicated that TID was an independent risk factor for disease progression, and was strongly associated with higher proteinuria, serum creatinine, and anti-PLA2R titers (79, 82).

These findings suggest that once interstitial fibrosis and inflammatory infiltration occur, they often indicate cumulative and irreversible renal damage. The physiological substrate for SR—viable podocytes and nephron units—is therefore significantly compromised. This supports prior observations that the more prominent the chronic histological changes, the lower the chance of disease reversibility. Thus, if biopsy reports reveal extensive tubular atrophy or interstitial fibrosis, clinicians should be cautious: even in cases with immunologic remission, full clinical remission may not be achievable, with persistent proteinuria in this context more likely reflecting background glomerular scarring rather than ongoing immune-mediated injury; therefore, additional immunosuppression is unlikely to be beneficial and management should prioritize maximal conservative therapy (renin–angiotensin system blockade, optimal blood pressure control, dietary sodium restriction, and potentially SGLT2 inhibitors—recognizing that evidence in MN is limited).

### 3.6 Demographic characteristics

Age and sex have also been implicated as potential predictors of SR in some studies. Classic natural history data suggest that younger patients (<50 years) and females tend to have higher SR rates. This may be due to differences in immune response intensity or better responsiveness to immunosuppressive therapy. Conversely, older male patients are often considered a higher-risk group. However, it is worth noting that MN overall has a male predominance, with an approximate male-to-female ratio of 2:1 (83–85). Therefore, demographic factors may be interrelated with other clinical characteristics. The impact of race on SR in MN remains unclear. While some studies suggest a higher incidence in White populations, there is no definitive evidence indicating significant differences in SR rates among different ethnic groups (85).

### 3.7 Other biomarkers

Several studies have explored additional biomarkers as potential predictors of SR. These include urinary selectivity index and the urinary IgG-to-α1-microglobulin ratio. Poor selectivity (i.e., high IgG excretion) is considered a marker of severe glomerular barrier damage and has been associated with worse prognosis (65). Genetic susceptibility (e.g., HLA-DQA1 alleles), degree of comorbid hypertension, and other clinical factors may also influence disease progression (86).

### 3.8 Lifestyle and modifiable risk factors

Individual patient factors and lifestyle choices can also influence the likelihood of SR. Excess body weight has emerged as an important consideration: observational data from glomerular disease cohorts indicate that obesity is associated with a significantly lower probability of achieving complete remission of proteinuria. In adults with nephrotic syndrome (including PMN), obesity was linked to about a 20–30% reduction in the hazard of remission (87). This may be due to obesity-related hemodynamic stress and inflammation, which can sustain proteinuria and kidney injury (88–90). Similarly, smoking has been identified as a modifiable risk factor that adversely affects renal outcomes. Smoking is a known accelerator of chronic kidney disease progression in glomerular disorders (91). In IMN, one cohort study found that current smokers had a dramatically higher risk of a 30% decline in eGFR compared to non-smokers (adjusted hazard ratio ~7–8), although smoking was not significantly associated with attaining remission in that particular analysis (92). Overall, smoking’s deleterious effects on the vasculature and immune response likely impede recovery; therefore, smoking cessation is strongly recommended in all MN patients. Current clinical guidelines emphasize lifestyle modifications — including weight control, exercise, and smoking cessation — as a cornerstone of supportive therapy (93). Indeed, the KDIGO 2021 glomerular disease guideline advises that all patients receive counseling on diet (e.g. salt restriction), quitting smoking, and maintaining a healthy body mass index as part of initial management (93). These measures not only improve general health but may enhance the kidneys’ capacity to stabilize or recover, thereby increasing the chance of SR.

Other patient-specific factors may play more subtle roles. For instance, the medications a patient takes can influence disease course. Certain drugs are known to exacerbate MN or cause secondary forms of it – for example, prolonged use of nonsteroidal anti-inflammatory drugs (NSAIDs) has been associated with the development or worsening of MN and nephrotic syndrome (94). In patients with PMN, the avoidance of nephrotoxic medications (such as NSAIDs) and other potential triggers is advised to prevent undue additional injury. On the other hand, adherence to supportive medications that treat comorbid conditions (like rigorous blood pressure control with renin–angiotensin system blockers) can facilitate reductions in proteinuria and promote remission. Additionally, “living conditions” encompassing factors like socioeconomic status, diet, and exposure to environmental toxins may indirectly affect SR. While direct evidence is limited, patients in favorable living circumstances — with good access to healthcare, ability to maintain proper nutrition, and minimal exposure to pollutants or infections — are theoretically more likely to have better outcomes. In contrast, poor living conditions or high levels of chronic stress could hinder overall health and delay renal recovery. Clinicians should therefore adopt a holistic approach: beyond the biochemical and histologic risk markers, addressing lifestyle and environmental factors (encouraging weight loss, smoking cessation, balanced diet, and medication review) is an integral part of maximizing the likelihood of SR in PMN.

Multiple factors collectively influence the likelihood of SR in MN. In general, low-risk features—such as female sex, younger age, healthy lifestyle factors, moderate baseline proteinuria, preserved renal function, negative or low-titer anti-PLA2R antibodies, and favorable early treatment response to conservative therapy (e.g., ACE inhibitors or ARBs)—are associated with a higher probability of SR. In contrast, high-risk features—including older age, male sex, obesity, smoking, heavy proteinuria, impaired renal function, and high-titer anti-PLA2R antibodies—indicate a lower likelihood of SR and may necessitate more aggressive therapeutic intervention. It is essential to emphasize that these factors represent probabilistic associations rather than deterministic outcomes. Individual patients may deviate from these patterns. Therefore, clinical decision-making should be based on comprehensive assessment and repeated dynamic follow-up to accurately evaluate disease trajectory and guide optimal management.

## 4 Treatment and management strategies

### 4.1 Clinical management of patients under observation

Given that a considerable proportion of MN patients may achieve SR, a “watchful waiting” strategy is commonly adopted for low-risk individuals. This approach involves withholding immunosuppressive therapy while providing intensive supportive care and close clinical monitoring (95). International guidelines, such as the 2021 KDIGO Glomerular Diseases Guideline, recommend stratifying patients by risk (low, moderate, high) before determining therapeutic plans (96). Patients presenting with proteinuria <3.5 g/day, serum albumin >30 g/L, eGFR >60 mL/min/1.73 m², and no major complications (e.g., AKI, infections, or thromboembolic events) are typically suitable for observation and supportive care. These patients should be followed regularly with assessment of anti-PLA2R antibody titers and clinical parameters.

**Figure 3:**
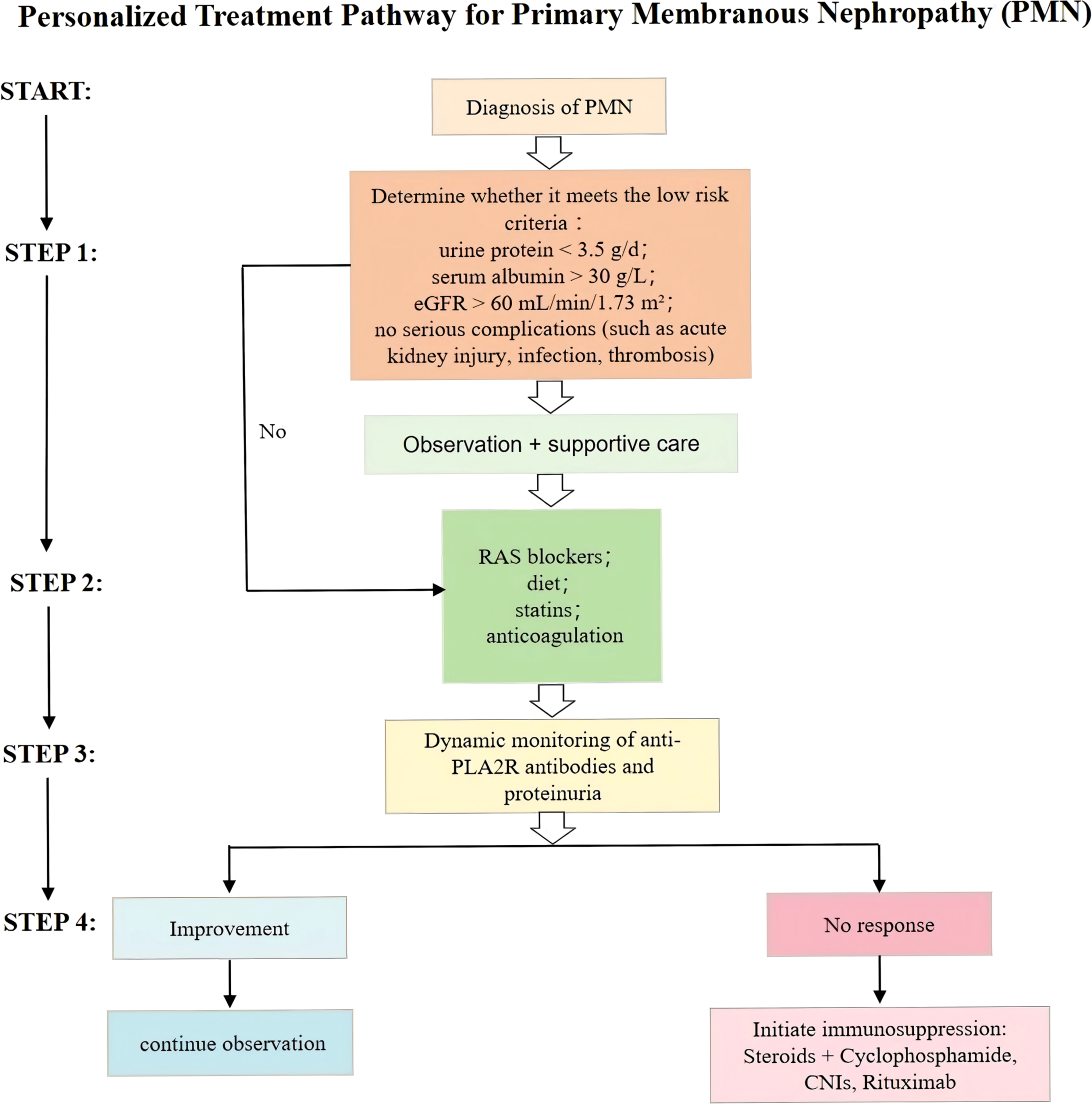
Personalized treatment pathway for primary membranous nephropathy (PMN) based on risk stratification, dynamic monitoring of anti-PLA2R antibodies and proteinuria, and stepwise therapeutic escalation.

However, if any of the following high-risk features are present—persistent heavy proteinuria (>4 g/day for >6 months), serum albumin <25–30 g/L, progressive decline in eGFR, persistently high anti-PLA2R antibody titers (>50 RU/ml, as per KDIGO guidelines), or serious complications such as AKI, refractory edema, or thrombotic events—immunosuppressive therapy should be considered. The goal of this strategy is to avoid unnecessary toxicity in those likely to undergo SR while ensuring timely intervention in patients at risk of irreversible renal damage (97, 98) (**Figure 3**).

Nevertheless, recent real-world data have raised concerns regarding prolonged conservative management in certain subgroups. A UK multicenter cohort study (99) reported that while low-risk patients achieved high SR rates (71%) with very low incidence of progressive CKD (9.5%) or ESKD (2.4%), moderate-risk patients with UPCR >600 — despite SR in approximately 30% (predominantly partial) — experienced substantial progression risk, with doubling of serum creatinine in 28% and ESKD in 16% over a median follow-up of 5 years. The mean eGFR decline of 7.7 mL/min/1.73 m² per year during watchful waiting highlights the challenge of balancing minimizing exposure/harm from immunosuppression (with hope of achieving SR) against loss of kidney function and progression to ESKD.

### 4.2 Role of non-immunosuppressive therapy in promoting remission

Supportive treatment forms the cornerstone of MN management and should be initiated promptly upon diagnosis to enhance the likelihood of SR and delay renal disease progression (7). Blood pressure control and anti-proteinuric strategies are central components of supportive therapy (100). Renin–angiotensin system (RAS) blockers, such as ACE inhibitors and ARBs, lower intraglomerular pressure and protein filtration, thereby providing established renoprotective effects in MN (101, 102). A multicenter study identified ACEI/ARB use as an independent favorable predictor of SR in MN (12).

In addition, sodium restriction and moderate protein intake (typically a high-quality, low-protein diet of ~0.8 g/kg/day) are recommended to reduce glomerular pressure and protein filtration burden, thus slowing renal function deterioration (103, 104). For patients with severe hypoalbuminemia and high thrombotic risk, individualized prophylactic anticoagulation may be considered based on bleeding risk assessment. Studies have shown that lower albumin levels are associated with a greater benefit-to-risk ratio for anticoagulation, particularly in patients with low bleeding risk. Decision-support tools incorporating both bleeding risk and albumin levels can help optimize anticoagulation strategies (105). Hyperlipidemia is commonly observed in MN, and statins may not only improve dyslipidemia but also offer potential benefits through anti-inflammatory effects and improvement of endothelial function (106, 107). Diuretics can be used to alleviate edema in MN patients; however, clinicians should be cautious of intravascular volume depletion due to excessive diuresis (108, 109).

Overall, through these comprehensive measures, supportive treatment alone can lead to a significant reduction in proteinuria, and some patients may even achieve partial remission without immunosuppressive therapy (12, 14). In practice, such “non-immunological” remission is often difficult to distinguish from true immunological SR, as the mechanisms of supportive therapy mainly involve improving hemodynamics and mitigating secondary injury (110), rather than eliminating immune complexes directly. Nevertheless, the adequacy of supportive therapy significantly influences disease progression, and it is critical to ensure that conservative management has been optimized before advancing to immunosuppressive treatment planning.

### 4.3 Monitoring during the observation period

For patients undergoing conservative management, close monitoring of parameters associated with disease activity and renal function is essential. Key clinical indicators include proteinuria, serum albumin, serum creatinine, and body weight (111–114). Dynamic changes in anti-PLA2R antibody titers offer important guidance for therapeutic decision-making. A progressive decline or seroconversion of anti-PLA2R antibodies during follow-up suggests attenuation of immunologic activity, indicating that the patient may be entering a phase of SR. In such cases, continued supportive care with vigilant follow-up is generally appropriate. Conversely, persistently elevated or rising anti-PLA2R titers reflect ongoing immunopathological processes and a lower likelihood of SR, warranting reconsideration of the treatment approach (4, 39).

### 4.4 Timing of immunosuppressive therapy

In MN patients who show no signs of improvement during the observation period or are classified as high risk, timely initiation of immunosuppressive therapy is essential to induce remission and prevent progression (115). The classical first-line regimen includes glucocorticoids in combination with alkylating agents (e.g., cyclophosphamide), which has been shown to significantly increase remission rates and improve renal outcomes (116, 117).

However, due to the potential toxicity associated with cyclophosphamide—including infection and malignancy—calcineurin inhibitors (CNIs) such as cyclosporine and tacrolimus, and anti-CD20 monoclonal antibodies such as rituximab, have increasingly been adopted as more tolerable alternatives (118). Several studies have reported that rituximab induces proteinuria remission in 60–80% of patients and is associated with a favorable safety and tolerability profile, making it an increasingly preferred first-line therapy in clinical practice (16, 17, 119). The need to initiate immunosuppressive treatment after a period of observation suggests that SR has not occurred or that the optimal window for intervention may have been missed. Therefore, immunosuppressive therapy should be initiated based on clearly defined clinical indications following an adequate observation period. The KDIGO 2021 guideline incorporates the concept of antibody-guided therapy and recommends shortening the observation period in patients with high anti-PLA2R antibody titers to optimize the timing of immunosuppressive intervention (18).

## 5 Future directions

### 5.1 Novel biomarkers

With advances in the molecular immunology of MN, the identification of novel biomarkers holds promise for improving the accuracy of predicting SR. First, new target antigens have been discovered in PLA2R- and THSD7A-negative MN cases, such as NELL-1, Sema3B, and EXT1/2 (120–122). To date, only anti-PLA2R and anti-THSD7A antibodies have been proven pathogenic in animal models. Clinically available assays are currently limited to anti-PLA2R ELISA (quantitative) and anti-THSD7A immunofluorescence (semi-quantitative). Antigen-specific MN subtypes may follow distinct clinical trajectories and remission patterns. For example, NELL-1-associated MN is characterized predominantly by IgG1 deposits and is occasionally associated with malignancies. Its disease course may differ from PLA2R-related MN (58). The development of clinically available assays for autoantibodies to novel antigens would facilitate prospective studies to correlate their levels with clinical outcomes, including SR, making them promising future biomarkers. Prognostic studies of these novel subtypes will help clarify their potential for SR and associated clinical behavior. In addition, detailed characterization of the autoantibody repertoire has garnered increasing attention. The epitope specificity of anti-PLA2R antibodies—i.e., the structural domains they recognize—has emerged as a key area of investigation (123). Studies have shown that the degree of epitope spreading at diagnosis is strongly associated with prognosis: patients with restricted epitope reactivity (e.g., only to the CysR domain) are more likely to achieve remission, while those with broader epitope recognition (e.g., CysR + CTLD domains) have poorer outcomes (41). Thus, epitope profiling of anti-PLA2R antibodies may become a valuable tool for risk stratification.

Other fluid-based biomarkers may also reflect disease activity. Recent research has identified PLA2R-rich migrasomes in the urine of MN patients, derived from podocytes. These may serve as natural urinary antigens for noninvasive monitoring, with potential utility in reflecting disease activity or remission status (124).

### 5.2 Predictive models for spontaneous remission

Multivariable predictive models represent a major direction for future research. Because single indicators are often insufficient to accurately predict individual outcomes, integrating clinical features with biological markers into comprehensive prediction tools may offer superior clinical utility. For example, one study developed a composite score incorporating both genetic risk and anti-PLA2R titers in PLA2R-related MN. The model outperformed clinical variables or antibody titers alone in predicting renal function decline, underscoring the value of multidimensional approaches (125). Another study involving 439 patients with idiopathic MN showed that a combination of age, eGFR, and proteinuria could be used to build a risk score with good predictive performance for renal function decline, end-stage kidney disease (ESKD), or death (126).

Future models may incorporate genetic polymorphisms (e.g., risk alleles in PLA2R1 and HLA-DQA1), as well as histopathological quantitative features such as the extent of glomerular immune complex deposition or IgG4 intensity (127, 128). With the application of machine learning techniques, it may be possible to develop scoring systems or risk calculators to predict SR probabilities, thereby offering clinicians objective and personalized tools for guiding patient management decisions. In line with this, findings from Hamilton et al. (99) highlight the urgent need for predictive tools capable of identifying patients at risk of significant renal decline during observation, particularly in moderate-risk groups. Such models, built on large datasets and validated across cohorts in different countries/populations, would be instrumental in optimizing the timing of immunosuppressive therapy and preventing the detrimental outcomes observed in this cohort.

## 6 Conclusion

Spontaneous remission represents a key clinical phenomenon in primary membranous nephropathy, offering a potential pathway to disease resolution without the risks associated with immunosuppressive therapy. Advances in our understanding of the immunological and structural underpinnings of SR—particularly the role of anti-PLA2R antibody kinetics, podocyte repair, and histologic reversibility—have refined our ability to identify patients likely to benefit from a conservative approach. Clinical predictors such as baseline proteinuria, renal function, and demographic characteristics, in conjunction with dynamic serological markers, can aid in individualizing therapy. As management strategies evolve, emphasis should be placed on optimizing supportive care and closely monitoring disease activity, reserving immunosuppression for those unlikely to remit spontaneously. Looking forward, the integration of emerging biomarkers, novel antigens, and predictive algorithms holds promise for enhancing prognostic accuracy and facilitating precision medicine in PMN.
